# Pathophysiology, symptoms, outcomes, and evaluation of hyponatremia: comprehension and best clinical practice

**DOI:** 10.1007/s10157-025-02624-9

**Published:** 2025-01-23

**Authors:** Hirofumi Sumi, Naoto Tominaga, Yoshiro Fujita, Joseph G. Verbalis, and the Electrolyte Winter Seminar, Collaborative Group, Hirofumi Sumi, Naoto Tominaga, Yoshiro Fujita, Takuya Fujimaru, Kazuhito Hirose, Kyogo Kawada, Toshiaki Monkawa, Masahiko Nagahama, Masatomo Ogata, Akihiro Ryuge, Yugo Shibagaki, Hideaki Shimizu, Maho Terashita, Masahiko Yazawa

**Affiliations:** 1 Division of Nephrology and Hypertension, Kawasaki Municipal Tama Hospital, 1-30-37, Shukugawara, Tama-ku, Kawasaki, Kanagawa 214-8525 Japan; 2 Division of Nephrology and Hypertension, Department of Internal Medicine, St. Marianna University School of Medicine, 2-16-1, Sugao, Miyamae-ku, Kawasaki, Kanagawa 216-8511 Japan; 3 Department of Nephrology, Chubu Rosai Hospital, 1-10-6, Komei-cho, Minato-ku, Nagoya, Aichi 455-8530 Japan; 4 Division of Endocrinology and Metabolism, Department of Medicine, Georgetown University, 4000 Reservoir Rd NW, Washington, DC 20007 USA

**Keywords:** Hyponatremia, Pathophysiology, Symptoms, Adverse outcomes, Evaluation

## Abstract

This review article series on water and electrolyte disorders is based on the 'Electrolyte Winter Seminar' held annually for young nephrologists in Japan. The seminar includes lively discussions based on cases, which are also partly included in this series as self-assessment questions. The first article in this series focuses on pathophysiology, symptoms, outcomes, and evaluation of hyponatremia, a common water and electrolyte disorder in clinical practice. Diagnosing the root cause(s) of hyponatremia can be challenging due to various etiologies and co-morbidities that affect water and electrolyte homeostasis, which can result in inappropriate management and worse outcomes in acute and chronic hyponatremia. This review provides an overview of pathophysiology, symptoms, outcomes, and evaluation of hyponatremia for better comprehension and improved clinical practice.

## Introduction

Hyponatremia is a water and electrolyte abnormality frequently observed in the general population [1] that often is not appropriately diagnosed or effectively treated. Clinicians face challenges in providing appropriate medical care for hyponatremia due to the following three reasons: (1) the need to perform differential diagnosis and treatment simultaneously, which is difficult in many cases; (2) insufficient established treatment strategies for various etiologies; and (3) inadequate testing for accurate diagnosis [2]. Hyponatremia can result from various etiologies, ranging from the most common such as the syndrome of inappropriate antidiuresis (SIAD) and medication-induced hyponatremia, to the less common such as adrenal insufficiency and salt-losing nephropathy. Besides these causes, multiple additional factors can contribute to development of hyponatremia, making it more difficult to determine the root cause.

Clinicians should promptly recognize hyponatremia in order to appropriately treat it, since not only acute moderate-to-severe symptomatic hyponatremia but also chronic mild symptomatic hyponatremia are associated with unfavorable outcomes. Therefore, this review details the pathophysiology, symptoms, adverse outcomes, and evaluation of hyponatremia to assist clinicians in clinical practice.

## Pathophysiology of hyponatremia

### Definition and prevalence of hyponatremia

Hyponatremia, defined as a serum sodium concentration ([Na^+^]) of < 135 mmol/L [3, 4], is classified into different categories according to the duration after disease onset (acute or chronic) and serum [Na^+^] (mild, moderate, or severe), respectively. It is also classified as no or minimally/mildly, moderately, or severely symptomatic based on the severity of hyponatremia-induced neurological symptoms, known as the 3-point symptom scale [5]. Specifically, all three classifications should be used to evaluate hyponatremia when diagnosed by clinicians (Tables 1 and 2) [4, 5].

**Table 1:** Definitions of hyponatremia (Modified from Reference [4, 5])

Definition by biochemical test value		Definition by duration from onset		Definition by severity of neurological symptoms	
Mild	130 ≤ , < 135 (mmol/L)	Acute	< 48 h	No or Minimal/Mild	Absence or presence of minimal/mild symptoms
Moderate	125 ≤ , < 130 (mmol/L)	Chronic	≥ 48 h (or unknown)	Moderate	Presence of moderate symptoms
Severe	< 125 (mmol/L)			Severe	Presence of severe symptoms

**Table 2:** Neurological symptoms of hyponatremia by severity (Reference [5])

Severity	Neurological symptoms
No or Minimal/Mild	Difficulty concentrating, irritability, altered mood, depression, unexplained headache
Moderate	Altered mental status, disorientation, confusion, unexplained nausea, gait instability
Severe	Coma, obtundation, seizures, respiratory distress, vomiting

Hyponatremia occurs in 14.5–42% of hospitalized patients and is associated with higher mortality [6, 7], though it has been suggested that the high mortality rate of hyponatremia originates more from the concomitant underlying comorbidities than from hyponatremia itself [8]. The risk of developing hyponatremia is also increased in patients with chronic kidney disease (CKD). An observational study involving Japanese patients with CKD revealed that the frequency of hyponatremia increased after CKD stage G3b [9, 10]. Reportedly, decreased body mass index (BMI) is one of the hyponatremia-associated factors [10]. In Japan, dietary guidance for patients with advanced CKD usually involves recommending solute intake restrictions, i.e., sodium, potassium, and protein intake restriction. Consequently, regarding the above-mentioned observational study on the prevalence of hyponatremia in CKD from Japan, we recently reported the possibility that restricting solute intake in patients with advanced CKD might have led to impaired renal free water excretion in addition to existing decreased renal function itself, resulting in an increased frequency of hyponatremia [11]. Based on the Edelman equation (Table 3) [12], hyponatremia in these patients might have progressed as the numerator [total body (exchangeable Na^+^ (Na^+^_e_) + exchangeable K^+^ (K^+^_e_))] decreased and the denominator [total body water (TBW)] increased. However, an observational study of United States (U.S.) veterans showed that the frequency of hyponatremia decreased from CKD stage G1 to G3a and did not change after G3b [13]. The difference in results between Japan and the U.S. suggests that hyponatremia is caused not only by impaired renal free water excretion resulting from CKD, but also by various other factors, including concomitant underlying diseases, diuretic use, and solute intake restriction.

**Table 3:** Formulae for hyponatremia (Reference [12, 18])

The Edelman equation	Serum [Na^+^] = 1.11 × Total body (Na^+^_e_ + K^+^_e_)/TBW – 25.6
P_Osm_ (mOsm/kg H_2_O)	2 × serum [Na^+^] (mmol/L) + BUN (mg/dL)/2.8 + glucose (mg/dL)/18
Effective P_Osm_ (plasma tonicity) (mOsm/kg H_2_O)	2 × serum [Na^+^] (mmol/L) + glucose (mg/dL)/18 or Measured P_Osm_ (mOsm/kg H_2_O)—BUN (mg/dL)/2.8
Urine tonicity (mOsm/kg H_2_O)	2 × urine ([Na^+^] (mmol/L) + [K^+^] (mmol/L))

*BUN* blood urea nitrogen, *K*^*+*^_*e*_ exchangeable potassium content, *Na*^*+*^_*e*_ exchangeable sodium content, *P*_*Osm*_ plasma osmolality, *TBW* total body water

### Difference between tonicity and osmolality

Dysnatremia is defined as an imbalance between (electrolyte-)free water and Na^+^ in the extracellular fluid (ECF). It is noted that when evaluating serum [Na^+^], total body Na^+^ and K^+^ should be considered. Specifically, hyponatremia and hypernatremia are states of absolute or relative excess or deficit of free water compared to Na^+^, respectively. Again, considering the Edelman equation (Table 3), serum [Na^+^] nearly equals total body (Na^+^_e_ + K^+^_e_) divided by TBW [12]. Free water movement occurs between the intracellular fluid (ICF) and ECF in dysnatremic states; therefore, understanding the difference between plasma osmolality (P_Osm_) and plasma tonicity (effective P_Osm_) is essential. The components of P_Osm_ include Na^+^, glucose, and urea, of which only urea can move freely between ICF and ECF. Effective P_Osm_ indicates the concentration of effective osmoles, with solutes being restricted to single fluid compartment, e.g., K^+^ inside and Na^+^ and glucose outside the cell. The tonicity between ICF and ECF is equal in principle; therefore, when extracellular tonicity changes, free water moves from the lower tonicity compartment to the higher tonicity compartment. Tonicity is defined as the force that moves free water osmotically between fluid compartments. Furthermore, extracellular tonicity is reduced in hyponatremia, and free water moves from the ECF to the ICF, resulting in cell swelling, whereas the opposite occurs in hypernatremia, which results in cell shrinkage.

Urine tonicity is mainly determined by Na^+^ and K^+^, but is not frequently measured in clinical practice despite its importance for evaluating pathophysiology of hyponatremia and determining best treatment options. Table 3 presents the equations for P_Osm_, effective P_Osm_, and urine tonicity. Serum [K^+^] can be omitted from the effective P_Osm_ equation because it is significantly lower than serum [Na^+^], while urine [K^+^] must be included in the urine tonicity equation because it is not significantly lower than urine [Na^+^].

### Mechanism of urinary dilution and concentration in the renal tubules

**Fig. 1:**
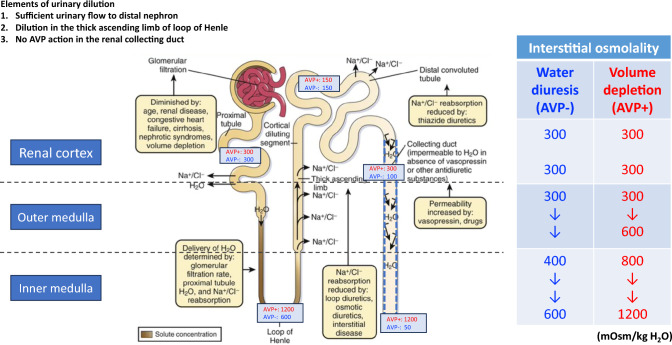
Elements of the urinary dilution mechanism and the conditions affecting them (Modified from Reference [14]). Three mechanisms are required for urinary dilution, and the pathophysiological conditions affecting these mechanisms are shown. The first mechanism requires sufficient urinary flow to the thick ascending limb of the loop of Henle as the diluting segment. This is influenced by decreased GFR due to age, CKD, and decreased EABV observed in heart failure, cirrhosis, and nephrotic syndrome. The second mechanism involves the reabsorption of solutes, i.e., Na^+^ and Cl^−^, in the diluting segment to supply diluted urine to the more distal nephrons, which is mainly influenced by loop diuretics and reduced reabsorption due to renal tubulointerstitial disorders. The third mechanism is the absence of AVP effects, which are influenced by SIAD or enhanced AVP effects produced by some medications. Additionally, when AVP secretion increases, such as during dehydration or ECF volume depletion, interstitial effective osmolality increases from the renal outer to the inner medulla (right table). The numbers in the tubular lumen in the figure represent urine osmolality in the renal tubular lumen at the corresponding site, depending on the presence or absence of AVP action. *AVP* arginine vasopressin, *CKD* chronic kidney disease, *EABV* effective arterial blood volume, *ECF* extracellular fluid, *GFR* glomerular filtration rate, *SIAD* syndrome of inappropriate antidiuresis

Hyponatremia is usually a state of relative excess free water that occurs when the ingested amount of free water exceeds the amount excreted through the urine and lost insensibly. In order to properly excrete free water in urine, the urine must be sufficiently diluted; hyponatremia may develop if this urinary dilution is impaired. The following mechanisms are necessary for urinary dilution: (1) sufficient urine flow to the thick ascending limb of the loop of Henle as the diluting segment; (2) reabsorption of solutes, i.e., Na^+^ and Cl^−^, at the diluting segment; and (3) absence of arginine vasopressin (AVP) action, also known as antidiuretic hormone (ADH) in the collecting ducts (Fig. 1) [14].

During dehydration or ECF volume depletion, urine becomes concentrated in the renal collecting ducts through free water reabsorption driven by the osmotic gradient between the interstitium and renal tubular lumen in the renal medulla. The two essential mechanisms for free water reabsorption are maintenance of a hypertonic interstitium in the renal medulla, and normal synthesis, secretion and receptor activation of AVP, which binds to and activates vasopressin V2 receptors (V2R) in the renal collecting ducts. V2R is G protein-coupled receptor that increases cAMP in renal collecting duct principal cells, with downstream effects to insert aquaporin-2 (AQP2) water channels into the apical (tubular) membrane, and promote transcription of AQP2 [15]. Sodium chloride (NaCl) and urea are essential solutes that maintain interstitial hypertonicity. Specifically, interstitial hypertonicity is maintained by NaCl reabsorption via Na–K-Cl co-transporters in the thick ascending limb of the loop of Henle in the renal outer medulla and urea reabsorption via urea transporter (UT)-A1/3 expressed in the renal inner medullary collecting duct [16]. AVP secreted during dehydration or ECF volume depletion also enhances UT-A1/3 expression [17]. Therefore, Na^+^ and Cl^−^ in the renal outer medulla and urea in the renal inner medulla are the primary solutes that cause interstitial hypertonicity.

## Symptoms and adverse outcomes of hyponatremia

### Acute hyponatremia

Acute hyponatremia causes intracellular edema due to the osmotic movement of free water into cells. As a protective mechanism against intracellular edema, electrolytes [including K^+^ (> 6–12 h)] and organic osmotic substances [including glutamine and taurine (> 24–48 h)] are extruded from cells to reduce intracellular edema [18]. Until these protective mechanisms are fully completed, (24–48 h), the brain is particularly susceptible to cerebral edema because the rigid skull limits expansion of the brain. Therefore, acute hyponatremia is usually associated with central nervous system symptoms (Table 2) [5]. Central nervous system symptoms resulting from cerebral edema are known as hyponatremic encephalopathy. Particularly, children, women, and patients with hypoxemia may be at higher risk of hyponatremic encephalopathy due to anatomical features and abnormalities in the protective mechanisms of the brain [19]. Exercise-related hyponatremia, synthetic narcotic 3,4-methylenedioxymethamphetamine ingestion, postoperative “aimless” administration of hypotonic fluids, and primary polydipsia are conditions that predispose patients to acute hyponatremia [18].

### Chronic hyponatremia

Blood tests are rarely performed within 48 h after hyponatremia onset in clinical practice; therefore, most cases should be considered and treated as chronic hyponatremia. Although patients with chronic hyponatremia often appear clinically asymptomatic, recent observational studies have suggested that hyponatremia may be associated with various adverse outcomes. Multiple chronic hyponatremia-associated adverse outcomes, including cognitive decline [20–22], gait instability [20, 23], falls [23–25], fractures [26, 27], osteoporosis [27–29], calcium kidney stones [30], and increased mortality rates [31], have been reported. Older patients with concomitant CKD or those taking medications that can cause hyponatremia are at risk of chronic hyponatremia, and efforts should be made to identify the cause of mild to moderate hyponatremia when detected.

## Evaluation of hyponatremia

**Fig. 2:**
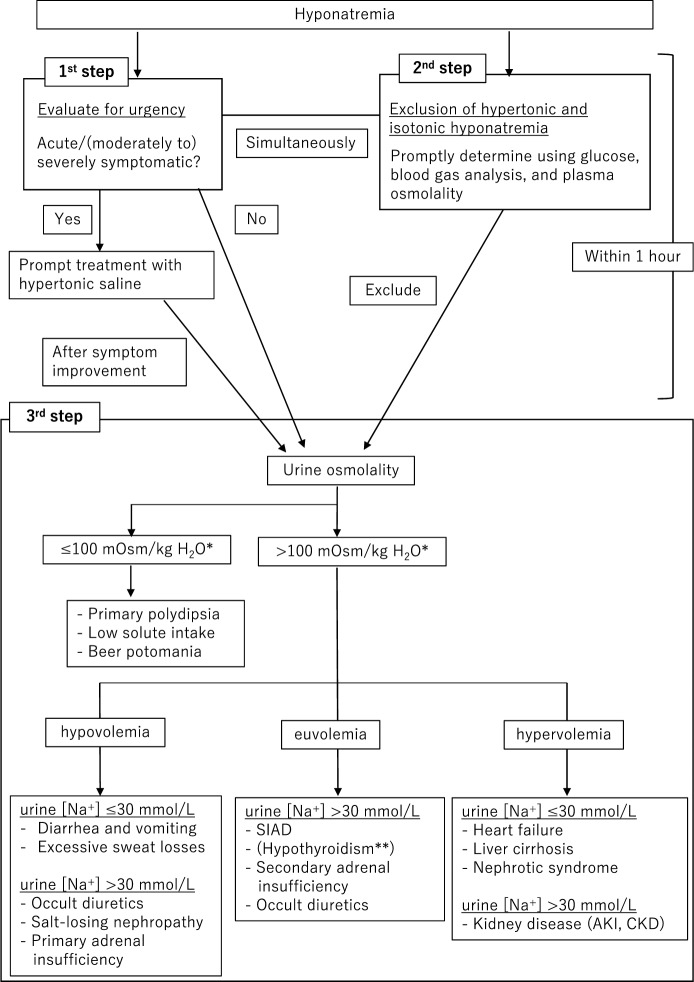
Algorithm for the treatment of hyponatremia. *Older individuals with age-related decreases in GFR may not be able to dilute urine to U_Osm_ < 100 mOsm/kg H_2_O, and a somewhat higher threshold (100–150 mOsm/kg H_2_O) might be appropriate. **Hypothyroidism does not impair urinary dilution unless it is severe (e.g., TSH > 50 μIU/mL), which generally only occurs with primary and not secondary hypothyroidism. *AKI* acute kidney injury, *CKD* chronic kidney disease, *GFR* glomerular filtration ratio, *SIAD* syndrome of inappropriate antidiuresis, *TSH* thyroid stimulating hormone

Several algorithms for differential diagnosis vary among existing hyponatremia guidelines. The representative guidelines, U.S. Expert Panel Recommendations, and European Clinical Practice Guideline have partly different algorithms; however, they commonly agree that isotonic and hypertonic hyponatremia should be ruled out before diagnosing hypotonic hyponatremia [3, 4]. Differentiating the etiology of hypotonic hyponatremia is usually challenging; consequently, it is typically diagnosed simultaneously with beginning treatment. Therefore, this review compiles these two algorithms and introduces them in a partly modified form that approaches diagnosis and treatment simultaneously (Fig. 2).

### Evaluation of hyponatremia: 1st step

Hyponatremia, when diagnosed, is first evaluated to determine whether the condition is emergent to treat. Urgency is determined sequentially as follows: (1) whether treatment should be started urgently based on neurological symptoms [5], or (2) whether the patient has progressive hyponatremia. Acute and (moderately to) severely symptomatic hyponatremia requires the urgent initiation of treatment. In these conditions, treatment must be initiated immediately, irrespective of the etiology of hyponatremia. Subsequently, it can be generally determined whether hyponatremia is likely to be progressive in case of no specific treatments by comparing the serum [Na^+^] and urine ([Na^+^] + [K^+^]). Specifically, this involves comparing the tonicity between glomerular filtrate and final excreted urine. The glomerular filtrate osmolality is usually equivalent to P_Osm_. Therefore, if blood urea nitrogen (BUN) and blood glucose concentrations are normal, the glomerular filtrate osmolality is approximated as 2 × serum [Na^+^], which equals the effective P_Osm_. In this context, a laboratory result that serum [Na^+^] is below urine ([Na^+^] + [K^+^]) indicates that the glomerular filtrate tonicity would be increased by reabsorbing free water by the time of the final excreted urine production. This indicates impaired renal free water excretion, i.e., impaired urinary dilution, and anticipated progression of hyponatremia. Conversely, if urine ([Na^+^] + [K^+^]) is below serum [Na^+^], hyponatremia will spontaneously improve due to the production of a large amount of dilute urine. It should be noted that hyponatremia will spontaneously and rapidly improve by removing the cause(s) of non-osmotic AVP secretion (e.g., ECF volume depletion, vomiting, and pain), even if urine ([Na^+^] + [K^+^]) is above serum [Na^+^] in cases of AVP secretion due to non-osmotic stimuli. Therefore, unless hyponatremic patients have moderate to severe neurological symptoms, hypertonic saline should not be used for the hyponatremia if the situation can change in a short time (e.g., ECF volume repletion, relief of vomiting or pain), since it may increase the risk of overly rapid correction of serum [Na^+^]. If neither (1) nor (2) is urgently determined, the next step involves differentiating hyponatremia.

### Evaluation of hyponatremia: 2nd step

#### Exclusion of hypertonic and isotonic hyponatremia

P_Osm_ should always be measured first to classify hyponatremia into hypertonic, isotonic, and hypotonic categories. Generally, P_Osm_ of > 295 and < 280 mOsm/kg H_2_O is defined as hypertonic and hypotonic hyponatremia, respectively, while isotonic hyponatremia falls in between these two ranges.

#### Isotonic hyponatremia

Isotonic hyponatremia has normal P_Osm_ despite hyponatremia, and it is sometimes mistakenly interpreted as pseudohyponatremia. It should be noted that isotonic hyponatremia includes not only pseudohyponatremia but also “translocational” hyponatremia. In isotonic hyponatremia, effective osmoles such as glucose, mannitol, sorbitol, glycine, and glycerol are present in the plasma, which increases the effective P_Osm_ causing free water to move from the ICF to the ECF, resulting in decreased serum [Na^+^]. This is termed a “translocational” hyponatremia, which is observed in hypertonic hyponatremia (see below) since it involves a shift of free water from ICF to ECF [4].

Pseudohyponatremia is a condition where the serum [Na^+^] appears low but P_Osm_ is in the normal range. It occurs when the amount of plasma water decreases due to high levels of solid components, including proteins and triglycerides, which occupy a portion of the total serum volume during the measurement. Consequently, the [Na^+^] per serum volume becomes low, whereas the [Na^+^] in serum water remains normal. Pseudohyponatremia occurs when measurement is performed using indirect potentiometry analysis and does not imply true hyponatremia. It is usually caused by hypertriglyceridemia or paraproteinemia induced by multiple myeloma. Therefore, treatment of the hyponatremia is unnecessary because the P_Osm_ is normal, and no free water movement from the ICF to the ECF occurs. However, if the diagnosis of pseudohyponatremia is uncertain, assessment should be conducted using direct potentiometry of serum [Na^+^] or blood gas measurements, which are unaffected by blood dilution.

#### Hypertonic hyponatremia

Effective osmoles, including glucose, mannitol, sorbitol, glycerol, and contrast agents, can move free water from the ICF to the ECF, causing translocational hyponatremia. Plasma hypertonicity occurs when the plasma concentrations of effective osmoles, which are the same contributors to isotonic hyponatremia, exceed the range that can be compensated for by free water movement from the ICF. Hyperglycemia lowers serum [Na^+^] by 1.6 mmol/L for every additional 100 mg/dL increase up to 400 mg/dL in blood glucose concentration from the normal concentration of 100 mg/dL, and by 4.0 mmol/L for every additional 100 mg/dL increase in blood glucose concentration from the concentration of 400 mg/dL if the blood glucose concentration is > 400 mg/dL [32]. Since this study reported an average decrease of 2.4 mmol/L, this value can be applied in clinical practice. Therefore, blood glucose concentrations should be examined in all patients with hyponatremia to accurately assess serum [Na^+^]. Moreover, assessing for recent administration of mannitol, sorbitol, glycerol, or contrast agents is necessary if the blood glucose concentration is normal.

Hyperosmotic hyperglycemic syndrome (HHS)-related hypertonic hyponatremia requires attention. In the therapeutic process of HHS, effective P_Osm_ is more important than P_Osm_; therefore, effective P_Osm_ should always be calculated during diagnosis and treatment (details will be presented in self-assessment question 1) [33]. P_Osm_ can be normal or elevated (> 280 mOsm/kg H_2_O) in the presence of azotemia, even if the pathophysiology is hypotonic hyponatremia. This is because of the discrepancy between P_Osm_ and effective P_Osm_ during azotemia, because urea nitrogen (UN) is not an effective osmole, although it is measured as a component of P_Osm_. Effective P_Osm_ decreases in hypotonic hyponatremia because the free water movements between the ICF and ECF are determined by effective P_Osm_ rather than by P_Osm_. Therefore, effective P_Osm_ should be calculated when azotemia is present, and a tonicity of < 280 mOsm/kg H_2_O should be treated as hypotonic hyponatremia.

### ~ Self-assessment question 1 ~

*Case 1:* A 50-year-old male patient presented to the emergency department with a fever. The patient was taking oral medications for type 2 diabetes. He had been sick for 2 days, with limited food and water intake. He experienced mild nausea and fatigue.

*Physical examination:* This revealed Glasgow Coma Scale (GCS) score = 13 (eye-opening, 4; best verbal response, 4; and best motor response, 5), blood pressure = 110/80 mmHg, pulse rate = 118 beats/min, oxygen saturation = 96% (room air), body temperature = 37.9 °C, and body weight (BW) − 2.0 kg compared with usual.

*Blood and urine tests:* This revealed serum [Na^+^] = 120 mmol/L, serum [K^+^] = 3.2 mmol/L, BUN concentration = 112 mg/dL, blood glucose concentration = 720 mg/dL, blood pH = 7.35, urine [Na^+^] = 21 mmol/L, urine [Cl^−^] = 18 mmol/L, urine [K^+^] = 29 mmol/L, urine osmolality (U_Osm_) = 800 mOsm/kg H_2_O, and urine ketones = 1 + .

Isotonic saline and regular insulin were initiated with an initial diagnosis of HHS. Blood tests 6 h later showed serum [Na^+^] = 130 mmol/L, BUN concentration = 56 mg/dL and blood glucose concentration = 360 mg/dL. No significant changes in vital signs occurred after treatment initiation; however, nausea and fatigue persisted.

#### Question

Which of the following is the MOST appropriate next step in treating hyponatremia in this patient?

A. Administer 5% dextrose (D5W)

B. Administer 3% saline

C. Continue isotonic saline administration

D. Measure blood gas

The correct answer is C.

Here, the P_Osm_ was calculated as 320 mOsm/kg H_2_O, indicating hypertonic hyponatremia. Compared to hypotonic hyponatremia, hypertonic hyponatremia usually results in a discrepancy between P_Osm_ and effective P_Osm_; therefore, effective P_Osm_ should be measured. In the abovementioned case, since urea does not form tonicity, the effective P_Osm_ was 280 mOsm/kg H_2_O at the presentation, and calculating P_Osm_ and effective P_Osm_ after 6 h showed 300 and 280 mOsm/kg H_2_O, respectively, revealing no change in effective P_Osm_. Generally, re-lowering to a lower serum [Na^+^] for overly rapid correction of hypotonic hyponatremia is to prevent osmotic demyelination syndrome (ODS) resulting from free water movement from the ICF to the ECF due to the rapid increase in effective P_Osm_. Additionally, A is incorrect because the effective P_Osm_ did not change, although the serum [Na^+^] was elevated, and re-lowering was not needed. Nausea and fatigue were present at the time of the visit and after 6 h, although the corrected serum [Na^+^] using the above-mentioned corrections for blood glucose concentration were approximately 135 mmol/L (120 + 2.4 × (720 − 100)/100 = 134.9) and 134 mmol/L (130 + 1.6 × (360 – 100)/100 = 134.2), respectively; therefore, hyponatremia-induced symptoms were unlikely to occur. Increasing the serum [Na^+^] is not urgently needed in HHS; therefore, B is incorrect. Although blood gas measurements help diagnose pseudohyponatremia, D is also incorrect because this case was a translocational hyponatremia, not pseudohyponatremia; little additional information would be obtained from blood gas measurements that would affect hyponatremia treatment. Severe ECF volume depletion occurs in HHS, and isotonic saline is recommended as the initial treatment. Therefore, if the corrected serum [Na^+^] is not elevated, the correct answer is C because isotonic saline would continue to improve P_Osm_ and blood glucose concentrations [34].

In 2024, the American Diabetes Association (ADA) consensus statement on hyperglycemic crises in adults with diabetes, published in 2001 [35] and last updated in 2009 [36], was updated as a consensus report [37]. In adults with HHS without renal or cardiac dysfunction, the administration of isotonic saline or balanced crystalloid at an initial rate of 500–1000 mL/h is recommended during the first 2–4 h to restore effective circulating plasma volume. Subsequently, fluid replacement therapy with isotonic saline or balanced crystalloid should correct estimated deficits within the first 24–48 h. However, caution should be exercised when fluids are rapidly replaced in those at high risk of ECF volume overload. Especially, older adults with HHS, as well as individuals with heart failure or end-stage kidney disease on dialysis, should be administered smaller boluses of isotonic saline or balanced crystalloid [38]. If HHS is present with no ketosis or with mild or moderate ketonemia and without acidosis, regular insulin should be administered at 0.05 U/kg BW/h intravenously. Conversely, if significant ketonemia representing mixed diabetic ketoacidosis/HHS is present, regular insulin should be administered at 0.1 U/kg BW/h intravenously [39].

### Evaluation of hyponatremia: 3rd step

#### Hypotonic hyponatremia

The differential diagnosis of hypotonic hyponatremia is usually difficult, even for nephrologists and endocrinologists, and it should be evaluated using multiple parameters. Various parameters are used for the differential diagnosis of hyponatremia, and we present a general method for differentiation using U_Osm_, urine [Na^+^], and ECF volume. U_Osm_ is calculated as 2 × urine ([Na^+^] + [K^+^]) + UN/2.8 and is generally regulated between 50 and 1200 mOsm/kg H_2_O. Measurement of U_Osm_ is necessary to determine whether impaired renal free water excretion causes or contributes to hyponatremia. Specifically, no disorder causing impaired urinary dilution exists if U_Osm_ is low; however, if U_Osm_ is not suppressed, a problem exists with urinary dilution. AVP regulates free water reabsorption in the renal collecting ducts. It is secreted in response to osmotic (increased effective P_Osm_) and non-osmotic stimuli, e.g., ECF volume depletion, medication-induced hyponatremia, and stress, such as pain, nausea, and vomiting, and concentrates urine by reabsorbing free water in the kidneys. AVP resistance (AVP-R, formerly called nephrogenic diabetes insipidus), is a urinary concentration disorder caused by reduced responsiveness to AVP due to vasopressin V2R dysfunction in the renal collecting ducts. However, a gain-of-function variant of the vasopressin V2R, which causes impaired urinary dilution despite plasma AVP concentrations below the detection limit, has been termed the nephrogenic syndrome of inappropriate antidiuresis (NSIAD) [40].

U_Osm_ of ≤ 100 mOsm/kg H_2_O implies that AVP secretion is suppressed and renal free water excretion is not impaired. Therefore, the cause of hyponatremia might be a relative or absolute excess of TBW due to polydipsia or insufficient solute intakes, such as in “tea and toast” syndrome or beer potomania. However, U_Osm_ of > 100 mOsm/kg H_2_O usually indicates the presence of impaired renal free water excretion. In this case, AVP secretion can be inappropriate for osmotic stimuli, but appropriate for non-osmotic stimuli. In hyponatremic patients with U_Osm_ of > 100 mOsm/kg H_2_O, ECF volume status and urine [Na^+^] further classifies hyponatremia. The U.S. Expert Panel Recommendations [3] and European Clinical Practice Guideline [4] differ in whether urine [Na^+^] or ECF volume status is evaluated first, but in clinical practice both should be evaluated together. Although fluid restriction is the cornerstone of hyponatremia treatment for euvolemic patients, not overlooking hypovolemic hyponatremia, which requires isotonic saline infusion during the early stages of treatment, is important.

#### Assessment of urine [Na^+^] and ECF volume status

Hypotonic hyponatremia is classified into hypovolemic, euvolemic, and hypervolemic hyponatremia based on ECF volume status. Determining the ECF volume status can be difficult unless the ECF volume changes are clinically apparent. Therefore, a combination of physical examination, blood and urine tests, imaging studies, and medical history should be used to differentiate the etiology of hypotonic hyponatremia. However, not delaying treatment of hyponatremia due to an excessive focus on differential diagnosis is important.

In hypovolemic hyponatremia, a urine [Na^+^] of ≤ 30 mmol/L indicates extrarenal Na^+^ loss, such as diarrhea, vomiting, and sweating, whereas a urine [Na^+^] of > 30 mmol/L indicates renal Na^+^ loss due to diuretics (natriuretics), salt-losing nephropathy, and primary adrenal insufficiency, among others. Additionally, a urine [Na^+^] of ≤ 30 mmol/L indicates heart failure, cirrhosis, or nephrotic syndrome in hypervolemic hyponatremia, whereas a urine [Na^+^] > 30 mmol/L may indicate renal failure, that is, acute kidney injury (AKI) and CKD. In any case, if the urine [Na^+^] is ≤ 30 mmol/L, the renin-angiotensin system is activated due to decreased renal blood flow (decreased renal perfusion pressure), resulting in increased Na^+^ reabsorption in the renal tubules. A urine [Na^+^] of > 30 mmol/L is usually associated with euvolemic hyponatremia. Most cases of euvolemic hyponatremia are SIAD; other differential diagnoses include secondary adrenal insufficiency, severe primary hypothyroidism, and occult diuretic use. Assessing whether the ECF volume status is normal is usually difficult in clinical practice; therefore, unless a clinically obvious ECF volume excess exists (e.g., peripheral edema, ascites), the response to a small volume of isotonic saline administration (generally 1–2 L) can be used to assess the ECF status. Specifically, in hypovolemic hyponatremia, isotonic saline administration suppresses appropriate and non-osmotic AVP secretion, and hyponatremia usually improves because of diluted urine excretion. However, euvolemic hyponatremia caused by SIAD usually does not improve with isotonic saline alone, and in some cases, this can worsen the hyponatremia. In the Hyponatremia Registry, 8% of patients with SIAD has a > 2 mmol/L decrease in serum [Na^+^] with isotonic saline [41].

### Common causes of euvolemic hyponatremia

#### SIAD/SIADH

**Fig. 3:**
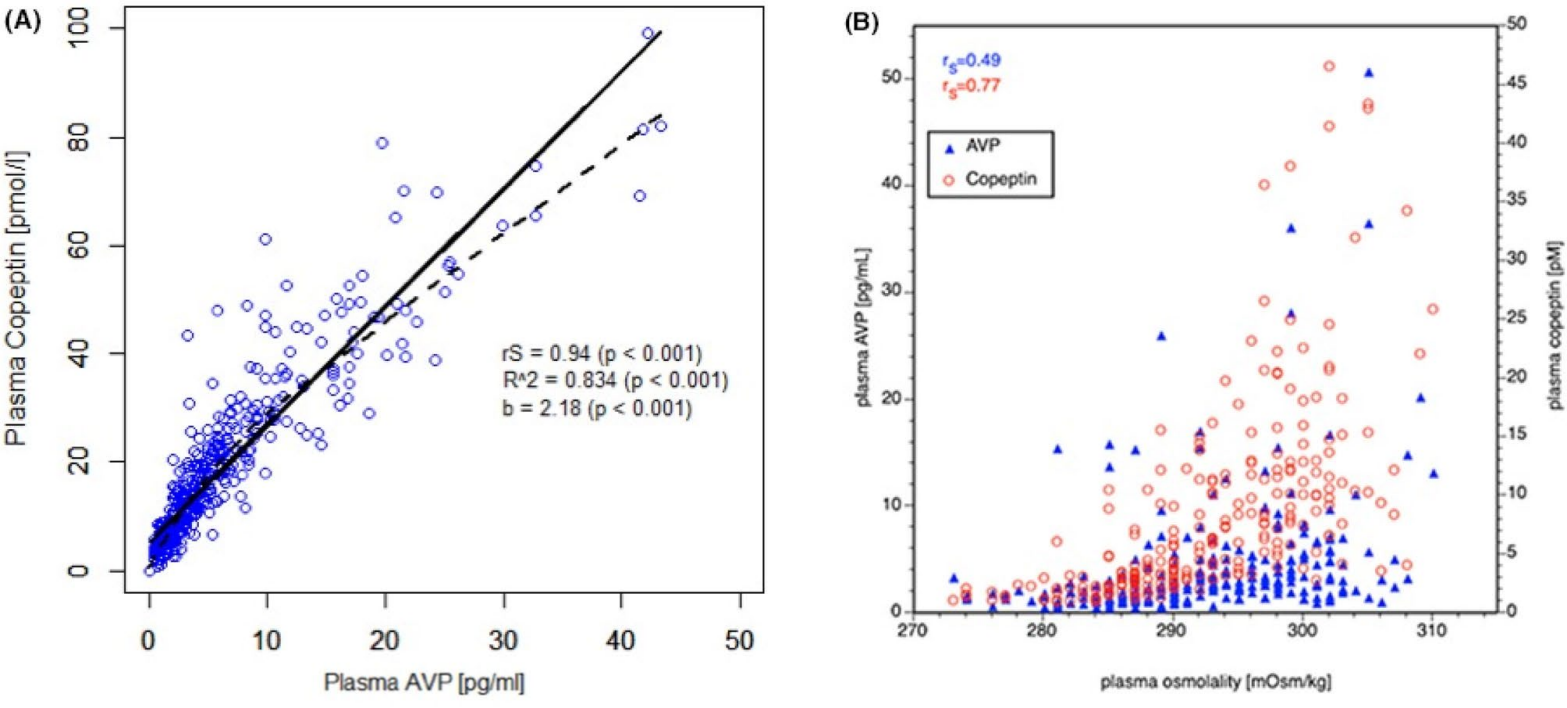
Correlation of AVP, copeptin, and plasma osmolality (Reference [49]). **A** Correlation between plasma AVP and copeptin concentration during osmotic stimulation by hypertonic saline administration. **B** Correlation between plasma AVP and copeptin concentrations when P_Osm_ increases from low to high osmolality. In both, a strong correlation was noted between the plasma AVP and copeptin concentrations. *AVP* Arginine vasopressin, *P*_*Osm*_ plasma osmolality

AVP is secreted from the posterior pituitary gland when P_Osm_ exceeds 285 mOsm/kg H_2_O, inserting AQP2 water channels into the luminal membrane of the renal collecting duct principal cells and promoting free water reabsorption, thereby preventing the development of hypertonic state such as hypernatremia. On the other hand, P_Osm_ of < 280 mOsm/kg H_2_O usually results in inhibition of AVP secretion [42]. Decreased effective arterial blood volume (EABV) and non-osmotic stimuli, including pain, nausea, and stress, cause AVP secretion independently of P_Osm_. AVP secretion in response to decreased EABV is actually appropriate for maintaining the ECF volume status. AVP is said to be “inappropriately” secreted in the syndrome of inappropriate antidiuretic hormone secretion (SIADH) despite the absence of osmotic stimuli and normal EABV by clinical evaluation. SIADH-like pathophysiology has been reported in patients even without detectable AVP secretion, therefore, “the syndrome of inappropriate antidiuresis (SIAD)” has been recommended to include patients who meet the Schwartz-Bartter criteria for this diagnosis [43, 44]. This terminology is preferred for several reasons, but mainly because 5–10% of patients with SIAD do not have measurable AVP secretion [45]. Consequently, SIAD and SIADH are clinically synonymous; therefore, strictly distinguishing between them is rarely necessary [46]. However, in the argument emphasizing the significance of AVP secretion and copeptin measurement, the use of “SIADH” would be still appropriate. In fact, the Japanese diagnostic criteria for SIADH require the plasma AVP concentration to be uninhibited despite hyponatremia or hypotonicity (Table 4) [47]. In this situation, plasma AVP measurements in Japan were suspended because the supply of antiserum used in the radioimmunoassay method, the only AVP measurement system in Japan, was temporarily discontinued. Copeptin is a hormone secreted into the blood by the cleavage of its precursor, pre-pro-vasopressin, into three peptides—AVP, neurophysin II, and copeptin—during axonal transport to the synapse in the posterior pituitary gland. It is secreted in an equimolar ratio to AVP and can be used to estimate the amount of AVP secreted by measuring copeptin concentration. Copeptin concentrations can also reflect detailed changes in AVP, whose secretion is immediately controlled in response to pathological changes (Fig. 3) [48, 49]. AVP measurements are again available for clinical use, while copeptin measurements are still limited to research purpose in Japan.

**Table 4:** Japanese diagnostic criteria for SIADH (Reference [47])

I. Main symptoms	No findings of ECF volume depletion
II. Laboratory data	1. Serum [Na^+^] < 135 mmol/L 2. P_Osm_ < 280 mOsm/kg H_2_O 3. Despite hyponatremia and hypoosmolality, plasma AVP concentration is not suppressed 4. U_Osm_ > 100 mOsm/kg H_2_O 5. Urine [Na^+^] > 20 mmol/L 6. Normal renal function 7. Normal adrenocortical function 8. Normal thyroidal function
Definitive diagnosis: all of I and II are met	

*AVP* arginine vasopressin, *ECF* extracellular fluid, *P*_*Osm*_ plasma osmolality, *U*_*Osm*_ urine osmolality, *SIADH* syndrome of inappropriate antidiuretic hormone secretion

Although central nervous system diseases, pulmonary diseases, malignancies, and medications are well-known causes of SIAD, the causative disease is sometimes not identified in clinical practice. More than half of SIAD cases, particularly in older patients, are reportedly diagnosed as idiopathic SIAD [50]. Diagnosis of SIAD is usually challenging because it is a diagnosis of exclusion; however, many reports have shown that serum uric acid (UA) concentration and fractional excretion of UA (FEUA) help differentiate SIAD from other causes of hyponatremia. This is because UA reabsorption in the renal proximal tubules is reduced in SIAD, resulting in hypouricemia [51]. Specifically, serum UA concentrations of < 4 and ≥ 4 mg/dL support SIAD and suggest decreased ECF volume, respectively [51]. An FEUA of > 10–12% supports the presence of SIAD, whereas that < 8% argues against SIAD [51, 52].

### ~ Self-assessment question 2 ~ 

*Case 2:* A 60-year-old male patient was treated with carboplatin-paclitaxel chemotherapy for primary lung cancer by a pulmonologist. Three days after the treatment initiation, nausea appeared, and mild hyponatremia with a serum [Na^+^] of 131 mmol/L developed. After 2 more days, the serum [Na^+^] decreased to 114 mmol/L. Isotonic saline was started as an initial treatment. However, the patient had no diluted urine excretion or improvement in hyponatremia; therefore, the pulmonologist consulted a nephrologist.

*Physical examination:* This revealed GCS score = 14 (eye-opening, 4; best verbal response, 5; and best motor response, 5), blood pressure = 126/84 mmHg, pulse rate = 70 beats/min, BW = 57.8 kg (unchanged), no orthostatic hypotension, and no pretibial edema.

*Blood and urine tests:*These showed P_Osm_ = 234 mOsm/kg H_2_O, serum [Na^+^] = 114 mmol/L, serum [K^+^] = 4.6 mmol/L, BUN concentration = 14.8 mg/dL, serum UA concentration = 2.6 mg/dL, blood glucose concentration = 98 mg/dL, plasma AVP concentration = 1.4 pg/mL (detection limit; 0.4), plasma adrenocorticotropic hormone (ACTH) concentration = 69.7 pg/mL (reference value; 7.2–63.3), plasma cortisol concentration = 21.7 μg/dL (reference value; 3.7–19.4), urine [Na^+^] = 97 mmol/L, urine [K^+^] = 57.9 mmol/L, and FEUA = 12.8%.

#### Question

Which is the MOST likely pathology of hyponatremia in this patient?

A. Dilutional hyponatremia

B. Isolated ACTH deficiency

C. Salt-losing nephropathy(associated with carboplatin administration)

D. SIAD

The correct answer is D.

The most likely pathology is SIAD, although it is usually difficult to diagnose since it is a diagnosis of exclusion. U_Osm_ should be examined first since this was a hypotonic hyponatremia case. The U_Osm_ was 673 mOsm/kg H_2_O, and A is incorrect since renal free water excretion was impaired. Since the cortisol concentrations were not low, B is incorrect, although several days are required to obtain plasma cortisol concentrations in clinical practice. Therefore, a comprehensive judgment is necessary based on the presence or absence of fatigue, weight loss, hypotension, eosinophilia, and other related symptoms until the results are obtained. The differentiation from salt-losing nephropathy is based on a combination of the ECF volume, Na^+^ balance, FEUA, and responsiveness to normal saline infusion [53]. In salt-losing nephropathy, the ECF volume typically decreases, and the Na^+^ balance becomes negative due to natriuresis. Some reports suggest that the failure of FEUA to normalize after hyponatremia treatment is a useful differentiator of SIAD [54]. Compared to SIAD, isotonic saline administration causes diluted urine excretion and improves hyponatremia in salt-losing nephropathy. This is because AVP secretion in salt-losing nephropathy is due to decreased ECF volume-associated non-osmotic AVP stimulation; when the ECF volume is corrected by isotonic saline administration, the non-osmotic AVP secretory stimulus disappears. Of note, if SIAD is complicated by subclinical ECF volume depletion due to another etiology, isotonic saline administration leads to diluted urine excretion, partial improvement in hyponatremia, and decreased serum UA concentrations [55]. In short, hyponatremia due to SIAD generally worsens with isotonic saline administration, however, hyponatremia due to SIAD with subclinical ECF volume depletion can partially improve with isotonic saline administration through suppressing physiologically appropriate AVP secretory stimulus for ECF volume depletion.

#### Medication-induced hyponatremia

Many drugs can cause drug-induced hyponatremia; however, natriuretics, antidepressants, antipsychotics, antiepileptics, and anti-neoplastic medications are the leading causes. Angiotensin-converting enzyme inhibitors (ACEi), proton pump inhibitors (PPIs), and nonsteroidal anti-inflammatory drugs (NSAIDs) are associated with hyponatremia, although at a low incidence [56]. The pathogenesis of medication-induced hyponatremia can be classified into mechanisms involving the relative amounts of free water and Na^+^ in the body and the factors that regulate body water and Na^+^ homeostasis. The typical agents associated with effects of body free water and Na^+^ are natriuretics, whereas those linked more with effects on body free water include antidepressants, antipsychotics, antiepileptics, antitumor medications, and NSAIDs. The effects on body water homeostasis include increased AVP synthesis in the hypothalamus, increased AVP secretion from the posterior pituitary gland, enhanced AVP action in the renal collecting ducts, and a reset osmostat, among others. Many drugs have been associated with increased AVP secretion, whereas some antiepileptics, antitumor medications, and NSAIDs are linked with enhanced AVP action. ACEi may also enhance AVP secretion by slowing bradykinin degradation [56]. The mechanism underlying rare cases of PPI-induced hyponatremia remains unknown; however, it may be related to both SIAD and salt-losing nephropathy [56].

###  ~ Self-assessment question 3 ~ 

*Case 3:* An 80-year-old female patient presented to an outpatient clinic of General Internal Medicine with fatigue lasting for 2 days. She had been prescribed a calcium channel blocker for hypertension, and a thiazide diuretic was added 2 weeks prior.

*Physical examination*: This revealed GCS score = 13 (eye-opening, 4; best verbal response, 4; and best motor response, 5), blood pressure = 145/68 mmHg, pulse rate = 82 beats/min, no decrease in skin turgor, no delayed capillary refilling time, and no BW loss.

*Blood test*: This showed serum [Na^+^] = 123 mmol/L, serum [K^+^] = 3.0 mmol/L, BUN concentration = 24 mg/dL, blood glucose concentration = 90 mg/dL, and P_Osm_ = 250 mOsm/kg H_2_O.

The patient was admitted to our hospital with a presumptive diagnosis of thiazide-associated hyponatremia (TAH). Therefore, the thiazide diuretic was discontinued, and serum [Na^+^] was normalized on the third hospital day.

#### Question

Which of the following is the MOST unlikely test result for this patient?

A. FEUA 15%
B. Serum UA concentration 2.4 mg/dL
C. U_Osm_ 100 mOsm/kg H_2_O
D. Urine [Na^+^] 70 mmol/L

The correct answer is C.

Hyponatremia in patients with a normal ECF volume by clinical assessment who are taking a thiazide diuretic is termed TAH, and includes cases even where thiazides are not the primary cause of hyponatremia. Thiazide-induced hyponatremia (TIH) is defined as TAH caused directly by thiazides. Fifty-four percent of patients with TIH (vs. 25% of controls) have at least 1 copy of a variant allele of *SLCO2A1*, which encodes a prostaglandin transporter (PGT) in the renal collecting duct [57]. Under normal conditions, the osmotic effects of increased AVP are offset by increased renal prostaglandin (PG) E2 production. The PGT directs PGE2 to the basolateral membrane of the renal collecting duct cells and promotes signaling through PG receptors 1 (EP1) and 3 (EP3), which inhibit AQP2 trafficking to the luminal membrane of principal cells thereby decreasing water reabsorption.

However, PGE2 concentrations in the lumen of the renal collecting duct increase when thiazide diuretics are administered to patients with *SLCO2A1* variants (reduced *SLCO2A1* activity), e.g., rs34550074 (p.A396T) [57]. Increased PGE2/EP4 signaling results in AQP2 insertion into the apical membrane and increases free water reabsorption, independently of AVP. However, the signal for the increased PGE2 production in this situation remains unknown [57]. A recent study showed that serum [Na^+^] are lower in individuals with higher urinary PGE2 and its metabolite (PGEM) excretion, whose association is pronounced in thiazide users in the general population. On the other hand, the single nucleotide polymorphism rs34550074 in *SLCO2A1* was not found to be associated with lower serum [Na^+^] or higher urinary PGE2 or PGEM excretion in thiazide or non-thiazide users in the general population. Based on these findings, PGE2-mediated renal water reabsorption may play a role in regulating serum [Na^+^], which is associated with the pathogenesis of both hyponatremia in the general population and clinically overt TIH [58].

Since TIH can only be diagnosed retrospectively (Table 5) [55], any hyponatremia that develops in a patient taking thiazide must be considered to be TAH in clinical practice. Therefore, thiazide discontinuation is essential in all patients with TAH [59]. However, a risk of overly rapid correction of hyponatremia exists due to abrupt discontinuation of the medication [60]. Typical TAH is more common in older females, develops within 2 weeks post-prescription although reports of hyponatremia onset even after 2 weeks exist, and has non-specific symptoms, including falls and fatigue. Although TAH pathogenesis remains unclear, excessive water intake, decreased body cations (Na^+^ and K^+^), and impaired renal free water excretion are the three associated mechanisms. Since the mechanism of pathogenesis includes impaired urinary dilution, C is incorrect because U_Osm_ is not low. TAH is frequently difficult to distinguish clinically from SIAD because the laboratory findings are usually similar on presentation: typical TAH usually results in high FEUA, low serum UA concentrations, and a urine [Na^+^] of > 30 mmol/L [55]. However, after dissipation of the natriuretic effects of thiazides (generally within 24 h after medication discontinuation), the urine [Na^+^] becomes < 30 mmol/L indicative of ECF hypovolemia.

**Table 5:** Criteria for diagnosis of TIH (Reference [55])

Euvolemia by clinical assessment
Improvement following cessation of thiazide treatment (by 3 mmol/L in 1 day or 5 mmol/L in 2 days)
No significant improvement before cessation of thiazide use (unless specifically treated with 3% saline, urea, or a vaptan)
No recurrence after resolution in the absence of a thiazide

*TIH* Thiazide-induced hyponatremia

#### Iatrogenic hyponatremia

**Fig. 4:**
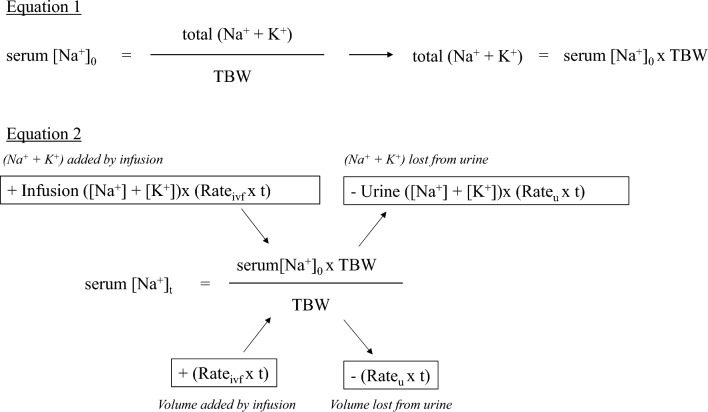
Prediction equation for correction of hyponatremia using the Edelman equation (Modified from Reference [62]). In Eq. 1, the total (Na^+^ + K^+^) in the body before treatment is calculated using the Edelman equation. In Eq. 2, the serum [Na^+^] after treatment is predicted if the volume of infusion and its composition, as well as the volume of urine and its composition, are known. For example, if a patient with serum [Na^+^]_0_ of 120 mmol/L, TBW of 50 L, and urine ([Na^+^] + [K^+^]) of 300 mmol/L is urinating at 50 mL/h, and isotonic saline [([Na^+^] + [K^+^]) = 154 mmol/L] is administered at 100 mL/h, we can calculate the predicted serum [Na^+^] after 12 h (assuming that the amount of urine and its composition remain unchanged). Substituting each into Eq. 2 yields 118.7 mmol/L. If the infusion fluid is hypertonic saline [for example, ([Na^+^] + [K^+^]) = 308 mmol/L], which is two times the concentration of normal saline, and is calculated under the same conditions, the predicted serum [Na^+^] is 122.3 mmol/L. Therefore, it is essential to administer infusions that are more hypertonic than urine ([Na^+^] + [K^+^]) to effectively increase the serum [Na^+^]. serum [Na^+^]_0_, Serum [Na^+^] before treatment; serum [Na^+^]_t_, Predicted serum [Na^+^] after t h; Rate_ivf_, Fluid volume per hour (L/h); Rate_u_, Urine volume per hour (L/h); TBW, Total body water (L) effectively increase the serum [Na^+^]

Iatrogenic hyponatremia is frequently observed in daily clinical practice, and should be guarded against because it can cause severe complications. The major causes are considered to be infusion of large volumes of fluids that are absolutely or relatively hypotonic to the patient's plasma, in concert with increased non-osmotic AVP secretion due to various causes. Although hypotonic infusions are usually implicated in iatrogenic hyponatremia, hyponatremia can occur or progress even when an isotonic ECF replacement solution with a higher [Na^+^] than serum [Na^+^] is administered. In the context of increased AVP secretion, particularly in the perioperative period, non-osmotic AVP secretory stimuli, including pain, nausea, and stress, easily lead to impaired free water excretion from the kidney. In this situation, urine tonicity [urine ([Na^+^] + [K^+^])] is usually higher than the ([Na^+^] + [K^+^]) of the ECF replacement solution, and the electrolyte component of the ECF replacement solution is excreted while the water component is retained (called “desalination” [61]). Consequently, serum [Na^+^] falls as part of the free water in the ECF replacement solution is reabsorbed, bringing the concentration of the administered ECF replacement solution closer to that of the final excreted urine [62]. Examining urine tonicity [urine ([Na^+^] + [K^+^])] to prevent iatrogenic hyponatremia, administering fluids with a higher tonicity than the urine tonicity, and reviewing the adequacy of fluid and enteral nutrition daily are necessary (Fig. 4). Chen et al. reported that infusions, even those with higher tonicity than urine tonicity [urine ([Na^+^] + [K^+^])], may decrease serum [Na^+^]; this is not usually a problem in clinical practice [62], but has been reported in 8% of patients with SIAD treated with isotonic saline [41].

## Conclusions

Hyponatremia varies among patients and frequently exhibits a heterogeneous pathophysiology, posing difficulty in diagnosing the cause. When hyponatremia is identified, it is crucial to evaluate for urgency based on neurological symptoms caused by the hyponatremia. The classification of neurological symptoms of hyponatremia known as the 3-point symptom scale (no or minimal/mild, moderate, and severe symptoms) mainly used in the U.S. is clinically relevant and also useful for treatment options. Once urgency is excluded, it is essential to evaluate the pathophysiology of the hyponatremia from multiple perspectives in order to perform appropriate treatment accordingly. We believe that this review will contribute to both comprehension of the pathophysiology, symptoms, outcomes, evaluation of hyponatremia, and application of this comprehension to guide best practices in the evaluation of hyponatremia.

## Data Availability

Not applicable.
